# Measurement of Wideband Absorbance as a Test for Otosclerosis

**DOI:** 10.3390/jcm9061908

**Published:** 2020-06-18

**Authors:** Lech Śliwa, Krzysztof Kochanek, W. Wiktor Jedrzejczak, Kacper Mrugała, Henryk Skarżyński

**Affiliations:** 1 Institute of Physiology and Pathology of Hearing, Mochnackiego 10, 02-042 Warsaw, Poland; l.sliwa@ifps.org.pl (L.Ś.); k.kochanek@ifps.org.pl (K.K.); k.mrugala@ifps.org.pl (K.M.); skarzynski.henryk@ifps.org.pl (H.S.); 2 World Hearing Center, Mokra 17 Kajetany, 05-830 Nadarzyn, Poland

**Keywords:** absorbance, middle ear, otosclerosis, tympanometry

## Abstract

The purpose of this study was to investigate the effectiveness of wideband energy absorbance in diagnosing otosclerosis by comparing the differences in acoustic absorbance between otosclerotic and normal ears. Exactly 90 surgically confirmed otosclerotic ears were included in the test group. The control group consisted of 126 matched normal-hearing subjects. The Titan hearing test platform (Interacoustics) was used for absorbance and acoustic immittance tests. Energy absorbance, measured at tympanometric peak pressure, was analyzed in the range 226–8000 Hz. Differences between normal and otosclerotic ears were analyzed in quarter-octave bands. Wideband absorbance, i.e., absorbance averaged over the 226–2000 Hz band, and resonance frequency were calculated and compared between normal and otosclerotic ears. Significant differences between the absorbance of normal and otosclerotic ears were found, especially at low and middle frequencies. No significant effect of ear side or gender was observed. For average wideband absorbance and resonance frequency, less pronounced (although significant) differences were found between normal and otosclerotic ears. Measurement of peak-pressure energy absorbance, averaged over a frequency band around 650 Hz, provides a valid criterion in testing for otosclerosis. The test is highly effective, with a sensitivity and specificity of over 85% and area under receiver operating characteristic curve above 0.9. Average wideband absorbance can also be used, but its effectiveness is lower. Other immittance-related measures are considerably less effective.

## 1. Introduction

Otosclerosis manifests in abnormal growth of the ossicular bones and bony fixation of the anterior stapes plate leading to stiffening of the chain. Its typical clinical symptoms are, among other things, progressive conductive or mixed hearing loss, increased or absent acoustic stapedial reflex thresholds (ASRT), and lack of otoacoustic emissions [1]. A comprehensive description of cases of otosclerosis, its symptoms, development and consequences, and methods of surgical treatment can be found in [2]. The gold standard for diagnosis of otosclerosis is intraoperative examination of the stapes and the ossicular chain. However, the decision about undertaking ear surgery is not unequivocal, as other middle ear problems (e.g., ossicular disarticulation and superior canal dehiscence) may have similar symptoms. Noninvasive methods of middle ear examination, such as imaging by high-definition computerized tomography (CT) or magnetic resonance imaging, still have insufficient resolution in detecting otosclerotic changes [3]. Thus, other noninvasive and easy test methods that could help qualify patients suspected of otosclerosis for stapedotomy (or other reconstructive ear operations) would be very useful.

Otosclerosis affects the mechano-acoustic properties of the middle ear, giving rise to alterations in immittance. The changes are the greatest in the low-frequency region, where middle ear impedance is dominated by stiffness [4]. Effects of otosclerosis can be understood by examining electric equivalent circuit models of the middle ear [5] or finite-element models [6,7,8]. Traditional low-frequency tympanometry can sometimes reveal changes in middle ear admittance due to otosclerosis (e.g., a decrease in static compliance), but in a significant percentage of cases the compliance of otosclerotic ears remains normal [9]. Multi-frequency multi-component tympanometry offers a more comprehensive insight into middle ear impedance and can help detect pathological effects. However, in practice it is troublesome to use and its sensitivity in detecting otosclerosis is still too low [10,11].

Acoustic immittance of the middle ear is measured by reflection of acoustic waves in the ear canal. The key quantities commonly used to characterize middle ear properties are wideband energy reflectance and energy absorbance. The latter expresses the ratio of energy absorbed by the middle ear to the total energy supplied to the ear canal. Energy reflectance is just the complement to the absorbance. Both are convenient measures, taking values between zero and one, and can be easily measured over a wide frequency range by supplying wideband stimuli (clicks or chirps) as test signals [12,13]. Compared with traditional tympanometry, measures based on absorbance or reflectance provide much more information about middle ear conditions and allow better evaluation of middle ear disorders [10,14,15,16,17,18].

Many investigations have confirmed that absorbance in otosclerotic ears is decreased compared to normal-hearing ears, especially in the low-frequency range [1,14,19,20,21]. The decrease is due to increased stiffness of the vibrating system which in turn gives rise to a decrease in low-frequency acoustic susceptance, and consequently an increase in reflection coefficient. At the same time, the resonance frequency of the middle ear system increases. Thus, an energy absorbance measurement (or other immittance-related measure) can be used as an indicator of otosclerosis.

The primary aim of the present research was to compare energy absorbance of otosclerotic ears to that of normal-hearing ears. To obtain reliable results, we included a relatively large number of patients with intraoperatively confirmed otosclerosis. The number was greater than those used in similar studies, and we employed a balanced control group. We also checked whether individual characteristics (laterality, gender, etc.) had an effect on absorbance, and examined whether other immittance-related measures, such as middle ear resonance frequency, could be used as useful criteria for detecting otosclerosis. To complete the picture, we formulated explicit numerical criteria for deciding on the presence of otosclerosis based on each of the immittance-related measures.

## 2. Experimental Section

All subjects gave written informed consent prior to participation in the study. The research procedures were approved by the Ethics Committee of the Institute of Physiology and Pathology of Hearing, Poland, approval No. IFPS:KB/20/2016 of 24 November 2016.

### 2.1. Clinical Procedures

All participants underwent comprehensive otologic and audiologic examination which consisted of clinical otoscopy and a battery of audiologic tests, including pure tone and impedance audiometry. In persons in the otosclerosis group, the condition of the middle ear was assessed intraoperatively during stapedotomy surgery.

Clinical audiometric assessment of each subject included air-conduction (AC) and bone-conduction (BC) audiometry. Clinical immittance tests consisted of standard 226 Hz tympanometry and ASRT measurements, followed by wideband absorbance measurements.

Otologic assessment included examining the ear canal and tympanic membrane by clinical otoscopy in all participants, and in patients from the otosclerosis group a comprehensive intraoperative assessment of the condition of the middle ear. The condition of the tympanic cavity and the shape, possible defects, and mobility of the ossicular chain were documented, concluding with a decision about the type of surgery required.

### 2.2. Instrumentation and Test Procedures

All audiometry tests were carried out in a sound-proof booth. All tests were performed within one day. In persons from the test group, it was a part of a pre-operative examination of them as a candidate for ear surgery.

Tonal audiometry was performed with a Madsen Astera clinical audiometer (Interacoustics). Hearing thresholds in air were determined at 0.125, 0.25, 0.5, 1, 2, 4, and 8 kHz, and for BC at frequencies of 0.25, 1, 2, and 4 kHz. For all ears the air–bone gap was calculated.

For impedance audiometry testing a Zodiac 901 Middle Ear Analyzer was used. Clinical immittance tests contained standard 226 Hz tympanometry and ASRT measurement using tone activators at 0.5, 1, 2, and 4 kHz in the ipsilateral mode, and the same tone frequencies in the contralateral mode. Activator level was limited to 110 dB SPL for tonal activators. The absence of an ASRT was marked as 100 dB SPL.

**Figure 1:**
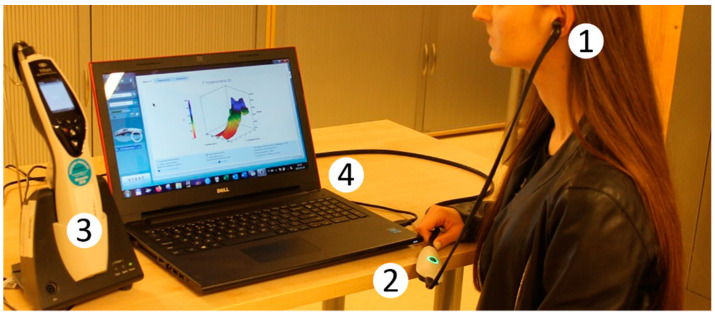
Measuring set-up for absorbance tests with Titan IMP440/WBT440 system. 1—Probe: presentation of stimuli and recording of acoustic signals from the ear canal; 2—Pre-amplifier with A/D converter; 3—Titan handheld unit: signal acquisition and processing; and 4—Titan PC suite with IMP440/WBT440 software: data analysis and OtoAccess^TM^ database.

For wideband absorbance measurements we used the software package (IMP440/WBT440 version v.3.2) for impedance and wideband tympanometry supplied with the Titan (Interacoustics) system, in the measurement set-up shown in Figure 1. Generally, the test procedures were used as recommended by the manufacturer. Both ambient-pressure absorbance and a set of variable pressure absorbances were measured; however, only the latter was used in this study. The measured frequency range was 0.226–8 kHz, with the ear-canal pressure varying from +200 to –300 daPa (downwards) at medium pump speed. The stimulus amplitude was typically about 100 dB peSPL (approx. 65 dB HL). The system allowed us to apply an extended test protocol that included upward and downward pressure sweeps, ambient-pressure measurement, and standard 226 Hz tympanometry. In selected cases the system was used for verification and comparison with other impedance audiometry tests.

**Figure 2:**
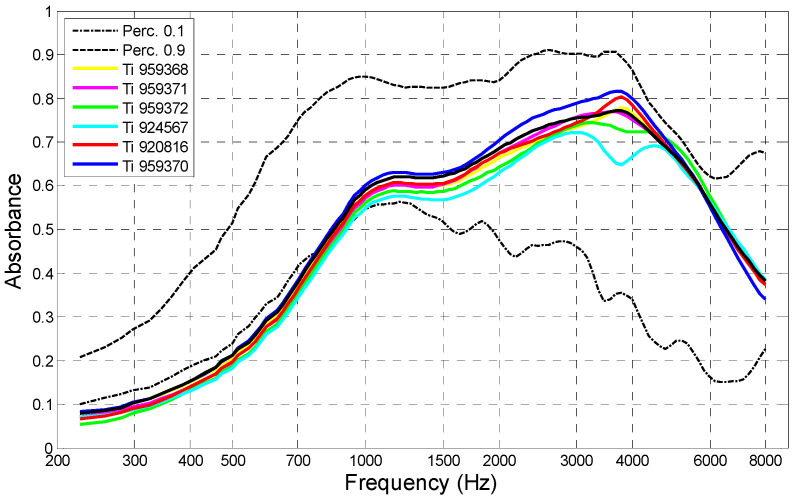
Absorbance–frequency characteristics of an artificial ear (BK4128C) measured with seven instruments used in the tests (Titan IMP440/WBT440). Dotted lines denote 10% and 90% absorbance limits for normal human ears.

The instruments were periodically calibrated in the manufacturer’s laboratories. A set of standard acoustic cavities was used to calibrate the source in order to set operating parameters. Additionally, before each series of tests, the stability and repeatability of the instruments’ performance was verified by measuring absorbance of an ear simulator (Brüel and Kjær head and torso simulator BK4128C). A set of absorbance–frequency characteristics of the ear simulator, measured with the seven instruments used in this study, is presented in Figure 2. The 10–90% absorbance range obtained from normal ears is also marked.

Measured absorbance data were extracted from the patient’s file (xml format) and frequency, pressure, and absorbance characteristics were stored in a numerical file. From the same source of data we extracted resonance frequency (FR), static compliance (SC) at 226 Hz, and other tympanometric parameters.

The Titan IMP440 instrument makes it possible to measure, among other things, the so-called wide-band absorbance, averaged over a certain frequency band. In the default test protocol, these bands are either 375–2000 Hz (for adults), or 800–2000 Hz (for testing children under 6 months of age). The bands, proposed by researchers who originally developed the method [16], are often applied in contemporary instruments. Among the reason for the choice of these frequencies is the fact that absorbance in this range is sensitive to middle ear pathology, and its variability is lower than at high frequencies. The plot of wide-band absorbance versus ear-canal pressure is called the wide-band tympanogram (WBT), which is an analogue to the traditional 226 Hz tympanogram. The pressure at which WBT attains its maximum is assumed to be the tympanometric peak pressure (TPP) in our research. For this pressure, the absorbance vs. frequency characteristic was recorded. In most cases, this pressure lay close (±5 daPa) to the tympanometric peak pressure determined by standard 226 Hz tympanometry.

It is known from other studies [16] that the TPP value depends on the direction of pressure change. Some authors recommend using two opposite pressure sweeps and, to eliminate bias, take the average. However, our previous work has shown that this bias is relatively small. We decided to use a downward sweep of pressure, as it is the default setting typically used in clinics.

It is known that one of the main sources of absorbance measurement errors is improper insertion of the probe in the ear canal (resulting in air leaks, etc.) [22,23,24,25,26,27]. To avoid such errors, we measured absorbance twice in each ear in the following series of four tests:

1. the probe placed in the first ear (randomly chosen);
2. the probe removed and placed in the contralateral ear;
3. the probe returned to the first ear, and the test repeated;
4. the test repeated in the ipsilateral ear.

We expected that satisfactory repeatability of results would be evidence that the probe was correctly placed in both sets of measurements. In such cases, an average of the two absorbance tests was calculated and stored as the final result. Repeatability of the absorbance–frequency characteristics was assessed by calculating the average discrepancy between the results, defined as the square root of variance between the tests averaged over the whole frequency range (it can also be interpreted as the average distance between the two traces). We stipulated that this error must not exceed 0.05. Excessive errors were rejected, or the tests were repeated in a following session.

### 2.3. Analyses

The data collected from the tests was used to determine the following: (i) the characteristics of absorbance vs. frequency, measured at TPP; (ii) SC at 226 Hz; and (iii) FR.

For statistical analyses, the interval 226 to 8000 Hz was divided into 21 quarter-octave bands with center frequencies of 241, 288, 343, 408, 485, 581, 696, 828, 985, 1172, 1393, 1657, 1971, 2344, 2787, 3315, 3942, 4688, 5575, 6630, and 7660 Hz. For each band the average absorbance was calculated, denoted AA241, AA288, AA343, and so on. We also calculated the average absorbance over the 226–2000 Hz interval, denoted AA2000, similar to that which is displayed in the Titan system as the peak value of the “wideband tympanogram”.

When comparing absorbances between the otosclerosis and control groups, we applied a mixed-model analysis of variance (ANOVA). In this model, gender (male and female) and group (norm and otosclerosis) were the between-subject factors, and ear (left and right) and frequency were the within-subject factors. Independent samples *t*-tests were used to compare absorbance, SC, and FR between the groups. Fisher’s exact test was also used to compare gender difference between otosclerotic and control groups.

To assess the accuracy of classifying ears as normal or impaired, we determined the sensitivity and specificity of tests based on different sets of criteria. The quantities considered were average absorbance in various frequency bands, SC and FR.

Receiver operating characteristic (ROC) and area under the ROC curve (AUC) were used to gauge the efficiency of the methods. An ROC curve plots the relative proportion of hits (sensitivity) against the number of false alarms (1—specificity). Sensitivity (SEN) is the likelihood of identifying an ear as impaired when hearing loss (e.g., otosclerosis) is present; specificity (SPE) is the odds of identifying a normal-hearing ear as normal; and efficiency is the proportion of ears that are correctly identified. AUC ranges from 0.5 for a test with no diagnostic power to 1.0 for a test with perfect diagnostic ability.

For analyses we used Statistica v. 12 (StatSoft Inc., Tulsa, OK, USA) and Matlab with Statistical Toolbox R2019b (MathWorks Inc., Natick, MA, USA).

### 2.4. Participants

Two groups of subjects were selected for this study: otosclerotic patients (the test group) and a cohort of normal-hearing persons (the control group). Later these groups are referred to as Otosclerotic and Normal.

The Otosclerotic group consisted of patients admitted to the Oto-Rhino-Laryngosurgery Clinic of the Institute as candidates for stapedotomy surgery. All patients were thoroughly examined before surgery, and otosclerosis was diagnosed based on clinical criteria: progressive conductive hearing loss, absence or abnormally high ASRT, absent otoacoustic emissions, and the results of otoscopic examination (in some cases, CT results were also available). The presence of otosclerosis was then confirmed intraoperatively during middle ear surgery [28]. All the patients in this group were provided with a middle ear prosthesis, in most cases the Kurtz–Skarzynski piston [2] (pp. 242–248), [29]. Assessment of the post-operative results from these patients, including audiometric and absorbance characteristics, will be the subject of another study.

From this group of patients, we excluded cases of (i) reoperation, (ii) multiple pathologies, and (iii) severe malformations of the middle ear.

The Normal group was recruited from volunteers. All candidates were comprehensively examined to confirm lack of hearing impairment. Inclusion criteria were: normal air-conduction hearing threshold level (≤20 dB HL in the 125–8000 Hz range), air-bone gap ≤10 dB from 0.5 to 4 kHz, ASRT <100 dB SPL, ear canal and tympanic membrane having no pathological changes or malformations as confirmed by otoscopic examination, and normal tympanometric characteristics at 226 Hz [9,30,31] (i.e., SC ≥ 0.3 mL; TPP within ±50 daPa; and type A tympanogram).

**Figure 3:**
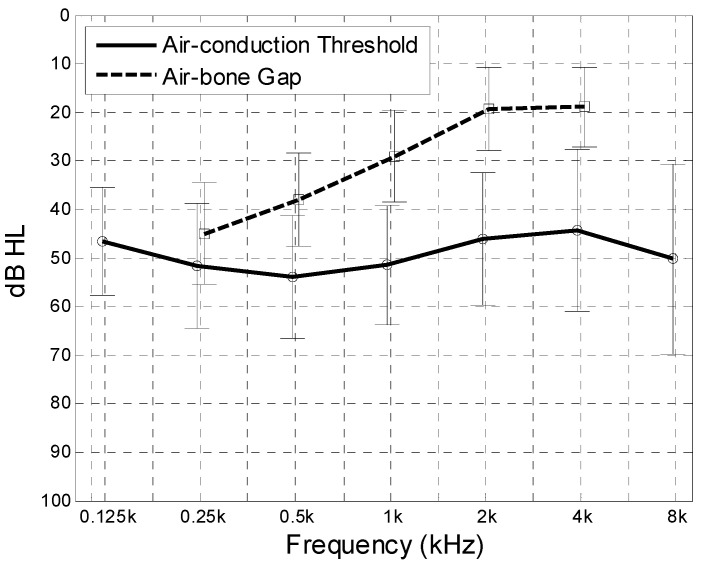
Preoperative air-conduction hearing threshold and air-bone gap in the Otosclerotic group. Mean value (thick lines) and standard deviation (whiskers).

Ages and genders of both groups are listed in Table 1, and preoperative audiometric characteristics of patients from the test group are shown in Figure 3.

As one might expect, the average age (both genders) in the Otosclerotic group was significantly greater than in the Normal group (*t*-test *p* < 0.0001). Also, there was a significant difference in age between men and women in the Normal group (*p* = 0.006); however, there was no such difference in the Otosclerotic group (*p* = 0.39). There was almost the same number of left and right ears in both groups, and a similar proportion of men to women (38/88 and 25/65 respectively). A *χ*^2^-test showed no significant difference between them (*p* = 0.704).

## 3. Results

Absorbance–frequency characteristics were measured in all subjects from both groups over a full range of ear-canal pressure, +200, …, –300 daPa, and frequency 226 Hz, …, 8000 Hz. Then, TPP was determined as described earlier, and the absorbance–frequency characteristic at this pressure was extracted for the following analyses. Additionally, we determined SC at 226 Hz and FR in all ears. All the data was extracted from the patient’s data file was stored in the Titan database.

**Figure 4:**
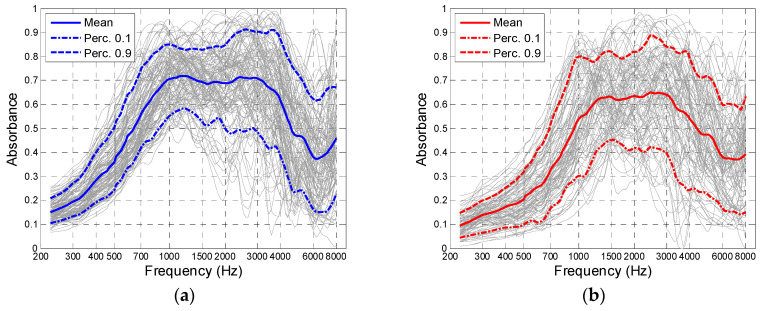
Absorbance peak-pressure against frequency for the Normal group (**a**) and Otosclerotic group (**b**). Solid lines mark the mean values and dotted lines mark the 10th and 90th percentiles.

**Figure 5:**
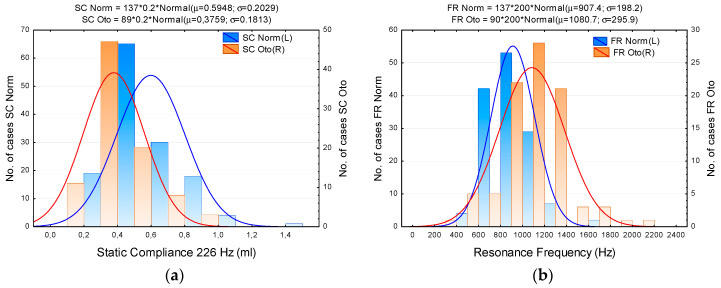
Histograms of static compliance (**a**) and resonance frequency (**b**) in the Normal (blue) and Otosclerotic (orange) groups. Key: SC Norm, SC Oto, FR Norm, FR Oto—Static compliance and resonance frequency in Normal and Otosclerotic groups, respectively; bars—number of cases in consecutive intervals, continuous lines—fitted normal distribution.

The following figures show absorbance against frequency for the whole Normal and Otosclerotic groups (Figure 4), or show histograms of SC and FR for these groups (Figure 5). Although the spread of the data is quite large, the differences in absorbance between Normal and Otosclerotic groups are easily visible when looking at mean and percentile values (Figure 4). In case of histograms of SC and FR (Figure 5), the distributions are quite similar and superimpose to some extent.

**Figure 6:**
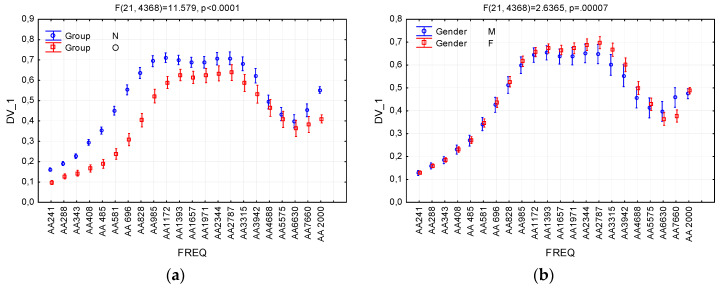
Effects of interactions between the variables Group (**a**), Gender (**b**), and absorbance, and the dependent variable DV_1. FREQ levels AA241, …, AA7660 denote average absorbance in consecutive quarter-octave frequency bands; AA2000 denotes average absorbance in the 226–2000 Hz band. Whiskers denote 95% confidence intervals. N—Normal group; O—Otosclerotic group; M—Male; and F—Female.

**Figure 7:**
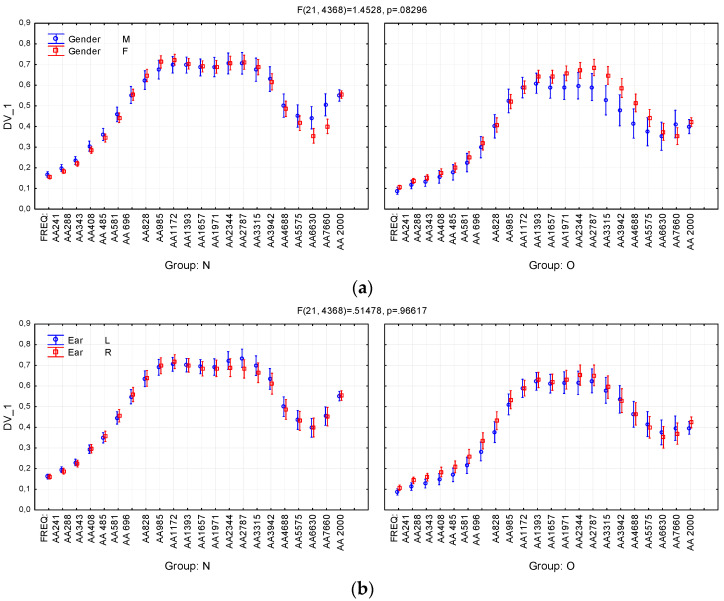
Effects of second-order interactions between the variables Group (N—Normal group; O—Otosclerotic group), Gender (M—Male; F—Female), Ear (L—Left; R—Right), and absorbance (the dependent variable DV_1). (**a**) Interactions between Group, Gender and absorbances; (**b**) Interactions between Group, Ear and absorbances. FREQ levels AA241, …, AA7660 denote absorbance averaged in consecutive quarter-octave frequency bands; AA2000 denotes absorbance average in the 226–2000 Hz band. Whiskers denote 95% confidence intervals.

In order to assess the effect of inter-subject and between-subject factors on absorbance, a repeated measures ANOVA model was applied, with ear (right vs. left) as within-subject factors, group (Normal vs. Otosclerotic) and gender (male vs. female) as between-subject factor, and frequency as repeated-measure factor (FREQ). Figure 6; Figure 7 illustrate the effect of these variables on absorbance (the dependent variable).

The only significant main effects were for Group (*F* = 119.2, *p* < 0.001) and FREQ (frequency) (*F* = 379.0, *p* < 0.001). Significant interaction effects were observed between FREQ and Group (*F* = 11.6, *p* < 0.001), FREQ and Gender (*F* = 2.64, *p* < 0.001), and Group and Gender (*F* = 6.37, *p* = 0.012). The remaining effects and interactions were below the level of significance. The complete results of ANOVA are listed in Table 2.

As can be seen in Table 2, the Group variable has the strongest effect on absorbance, and significant differences between Normal and Otosclerotic groups appear in most of the analyzed frequency bands. It can also be seen in the Figure 6a that there is a very strong effect size of Group. Figure 6b shows a moderate (but significant) effect of the Gender variable, which becomes clearer when one takes into account second-order interaction between Group, Gender, and FREQ (Figure 7a). The effect is clearly visible in the Otosclerotic group for instances of FREQ between AA1393 and AA5575; however, there is no such an effect in the Normal group. Similarly, the second-order interaction between Group, Ear, and FREQ variables is negligible (Figure 7b).

To quantitatively assess the differences between the Normal and Otosclerotic groups, a nonparametric Mann–Whitney U-test was applied. The results make it possible to evaluate also the AUC (area under ROC curve) of a test that might be applied for detecting otosclerosis based on absorbance as the test criterion. AUC is a convenient and widely used measure for assessing the effectiveness of a test [32,33]. AUC values are shown in the last column of Table 3.

As shown in Table 3, significant differences between the two groups exist (*p* < 0.05) in almost all considered bands, except for AA4688, AA5575, and AA6630. The greatest differences (maximal *U*, *Z*, and AUC values) appear in some low and medium frequency bands (AA408–AA828), and in AA2000 (absorbance averaged in the 226–2000 Hz interval). In all these cases, AUC takes maximum values of ≥0.86. It therefore seems reasonable to use absorbances from these bands as a criterion for otosclerosis.

According to the above results, the most useful measure would be absorbance averaged in the frequency interval encompassing the range of maximum AUC, i.e., the 545–749 Hz band. Average absorbance in this band will henceforth be denoted AA650. Other immittance-related measures that might be taken into account as test criteria are AA2000, SC, and FR.

Table 4 presents results of Mann–Whitney U-tests comparing these measures between Normal and Otosclerotic groups.

**Figure 8:**
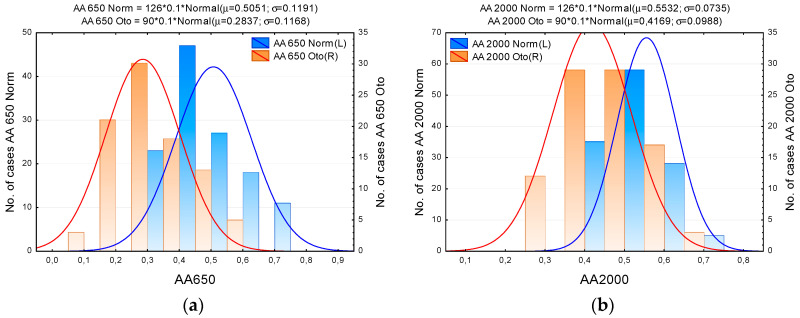
Distributions of average absorbance over the 545–749 Hz (AA650) and 226–2000 Hz (AA2000) bands in the Normal and Otosclerotic groups. (**a**) Histogram of absorbance AA650; (**b**) Histogram of absorbance AA2000. Key: AA650 Norm, AA650 Oto, AA2000 Norm, AA2000 Oto—Absorbance in Normal and Otosclerotic subjects, respectively. Bars represent number of cases in consecutive intervals. The lines show fitted normal distributions.

The histograms in Figure 8 show distributions of AA650 and AA2000 values in the Normal and Otosclerotic groups. Again, as in previous figures, there is quite a large spread of the data but it is evident that the separation of the groups is better for AA650.

**Figure 9:**
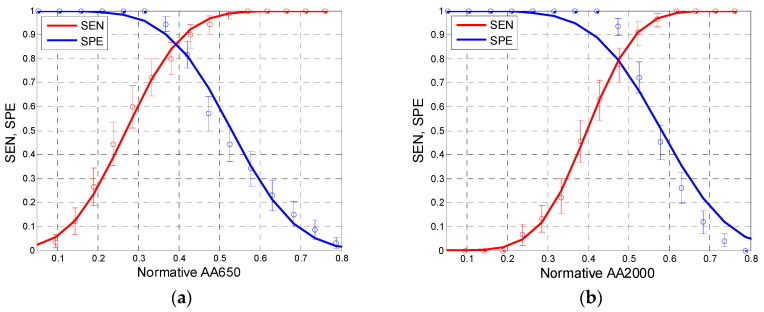
Sensitivity (SEN, red) and specificity (SPE, blue) of tests based on absorbance values for AA650 (**a**) and AA2000 (**b**) as test criteria. Lines show approximations to normal distribution functions (based on data from Figure 8). Whiskers denote 5–95% confidence intervals.

**Figure 10:**
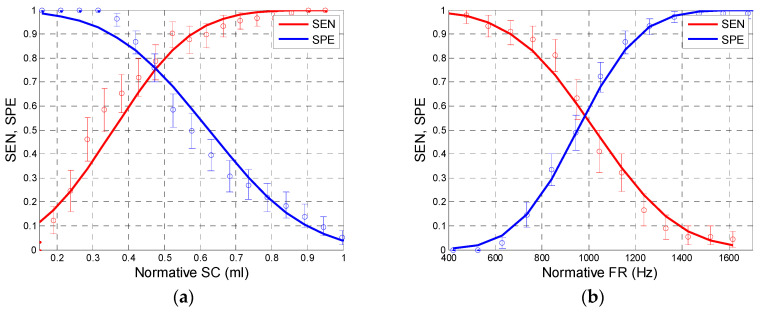
Sensitivity (SEN, red) and specificity (SPE, blue) of tests based on static compliance (SC) (**a**) and resonance frequency (FR) (**b**) as test criteria. Lines show approximations to normal distribution functions (based on data from Figure 5). Whiskers denote 5–95% confidence intervals.

Based on the distributions in Figure 5 and Figure 8, we can determine approximate sensitivity–specificity functions for the tests in which AA650, AA2000, SC, and FR are used as test criteria. The results are shown in Figure 9 and Figure 10. Although the size of our examined sample was relatively large (a total of 216 ears, including 90 confirmed cases of otosclerosis), the characteristics (*SEN, SPE*) determined experimentally in that sample might differ from the “true” values determined in the entire population [34]. To assess the range of uncertainty, we calculated confidence intervals for sensitivity and specificity. It is usually assumed [35] that both *n*_1_*p*_1_ (= *n*_1_⋅*SEN* = *TP*) and *n*_2_*p*_2_ (= *n*_2_⋅*SPE* = *TN*) have independent binomial distributions *n_i_p_i_* ~ B(*n_i_,p_i_*), where *i* = 1, 2 and *n*_1_, *n*_2_ are the numbers of impaired and normal ears in the population sample, respectively; *p*_1_, *p*_2_ stand for the sensitivity and specificity of the test; and *TP*, *TN* are the numbers of true positive and true negative test results. We calculated (0.05, 0.95) confidence intervals for *TP* and *TN* directly from the appropriate binomial distribution functions. As Figure 9 and Figure 10 show, the confidence intervals are not quite symmetrical; nevertheless, the approximating curves of SEN and SPE lie within these intervals.

As these figures show, the courses of the sensitivity and specificity functions differ depending on the applied test criterion. The best performance of test is achieved at the intersection point of sensitivity and specificity values. We may estimate these values as approximately 0.86 for AA650, 0.8 for AA2000, 0.75 for SC, and 0.55 for FR.

**Figure 11:**
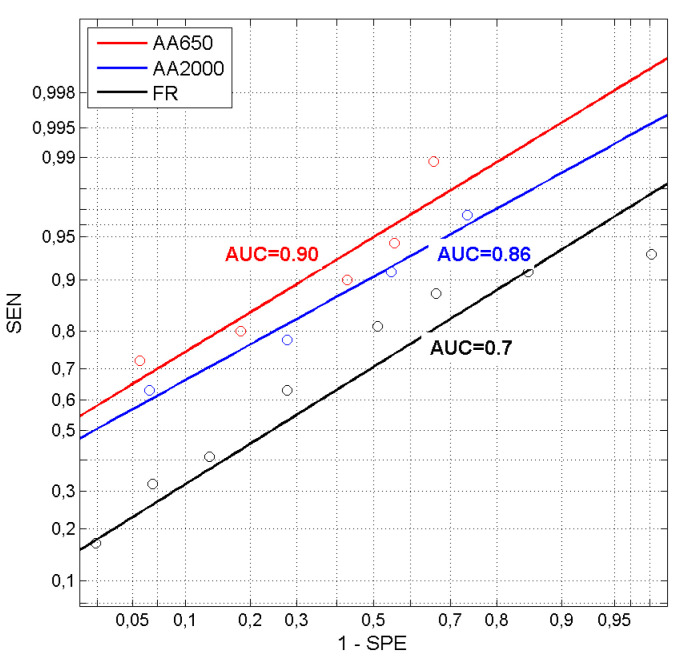
Receiver operating characteristics for tests based on average absorbance for AA650 and AA2000 and resonance frequency (FR) plotted in units of inverse normal distribution (*z*-scores). Points in the graph are actual experimental values and the lines show a linear approximation of ROC curves. AUC values for each curve are also shown.

Figure 11 shows the course of ROC curves for different test criteria. The graphs are plotted in coordinates of cumulative normal distribution functions Φ^–1^ (1—SPE) and Φ^–1^ (SEN) [33].

For normally-distributed test results of true-positives and false-positives, a graph of SEN vs. (1–SPE) plotted in nonlinear coordinates of units of inverse normal distribution would take the form of a straight line [32]. If its position above the coordinate center (intersection of (0.5, 0.5) coordinates) is related to the value of AUC, then one can graphically evaluate the effectiveness of a test. Actual test results, the points shown in Figure 11, are scattered around the approximating regression lines, which means that their distribution is not strictly normal. However, even the approximation lines clearly show which of the tests is better, and allow one to estimate its AUC value. As seen in Figure 11, the test based on AA650 is superior over the remaining ones (the line for AA650 is above other lines).

We have also examined relationships between the applied immittance-related measures. Table 5 shows cross-correlations between AA650, AA2000, SC, and FR in the Normal and Otosclerotic groups.

As one might expect, correlations between all these variables are significant (*p* < 0.05) with correlation coefficients *r* in most cases above 0.5, which denotes a large effect size.

## 4. Discussion

The aim of the present research was to find a method, easy to use in a clinic, for testing for the presence of otosclerosis. For that reason, we decided to use a commercially available instrument (i.e., the popular Titan WBT Immittance System) and rely on typical test protocols to measure immittance-related quantities. Unlike some other similar studies [1,16,17,36,37,38], we did not want to use dedicated systems made for research purposes. We wanted data for analyses to be easily obtained directly from the Titan system. The only exception was the average absorbance in selected frequency bands. However, this can be obtained by simple calculations based on the system data.

It is known that experimentally determined absorbance (as well as other similar measures) exhibit significant variability, both in normal hearing subjects [39] and in pathological ears [1]. This is due to natural inter-subject variability of ear acoustic properties [22] and random measurement errors of diverse origin [23,24,26]. As a result, the “normal” and “pathologic” range of absorbance values largely overlap, and this makes it difficult to distinguish between norm and otosclerosis. 

Assessment of the effectiveness of various tests for otosclerosis based on immittance measures has been the subject of a number of studies [1,10,14,15,19,36,40,41,42]. Authors have considered: (i) ambient pressure or peak pressure absorbance average in selected bands (typically 200–2000 Hz) [1]; (ii) resonance frequency (or frequency at *π*/2 phase shift) of the middle ear admittance [11,14]; and (iii) the group delay of energy reflectance [1,39]. It has been found that all of them have limited effectiveness, and do not provide adequate sensitivity and specificity for otosclerosis testing. It is also generally known that static compliance (absolute admittance value at 226 Hz) is the least suitable measure for this application [11,14,30].

In order to achieve the above-mentioned aim of the research, we decided to eliminate sources of errors as much as possible and look for alternative measures that could be used as test criteria. The accuracy of the instrumentation was carefully checked, instrument readings were verified with the use of an admittance standard (artificial ear), and we ensured that testers had undergone practical training on a group of healthy volunteers before they started examining patients. To limit errors resulting from improper insertion of the probe in the ear canal, we instructed the testers to repeat the test twice, each time re-inserting the probe from ipsi- to contralateral ear. Absorbance from two insertions in the same ear were compared and a consistency measure was calculated based on the average variance between two tests. In this way, we were able to obtain reliable results in the majority of examined ears. Only in a small number of cases (less than 5%) did we get outliers, and these were identified as being due to problems with non-cooperative patients, additional ear problems such as fluid in the tympanic cavity, cholesteatoma, etc., and these results were neglected.

Personal characteristics of both examined groups (Normal and Otosclerotic) were compared statistically. The greater number of women in the Otosclerotic group results from the greater prevalence of otosclerosis in females [29]. There was a significant difference between the mean age of subjects in the two groups. Because otosclerosis develops in older adults, these persons were generally older than the normal-hearing subjects recruited to the control group (the Normal group). The younger age of the control group reflects a practical reason: it is difficult to collect enough healthy normal-hearing persons of mean age of about 50. The age difference might have some effect on the immittance measures; however, there are no reports that confirm age-related changes in absorbance in healthy ears [43].

In order to analyze the impact of otosclerosis on frequency characteristics, as well as the effect of personal characteristics in both groups, we applied a repeated-measures ANOVA model, taking “group” and “gender” as inter-personal factors, “ear” as the intra-personal factor, and frequency as the repeated-measure factor (in the analyses here called FREQ). The analysis showed very significant effects of “group” and frequency, which is already well understood (see Figure 6). There was also an interaction of “gender” with “group”, so that a significant difference between genders appeared at high frequencies (2–6 kHz) in the Otosclerotic group. We find it difficult to explain this effect, and it is not one that has been confirmed by other researchers. Further investigations are needed to verify if absorbance really is higher in otosclerotic women in this region. In practical terms, however, this phenomenon is not important because our preferred test relies on absorbance in a different frequency range (below 2000 Hz).

Our results show the greatest differences in absorbance between the groups in the low and medium frequency range, up to 2000 Hz. It therefore makes sense to investigate this region and examine whether there are any measures that might be used as a criterion for otosclerosis. Because the distributions of absorbance values, in both groups and in practically all frequency bands, do not fulfill the criteria of normality, we applied a non-parametric Mann–Whitney U-test to compare groups. The test confirmed significant differences at almost all frequencies (except in the 4.7–6.6 kHz range); moreover, it allowed specific AUC values of tests for otosclerosis to be calculated. The larger AUCs, above 0.9, appear in the frequency range of approximately 550 to 700 Hz, so we decided to take the absorbance averaged over this band, denoted AA650, as a test criterion for further investigation. The other quantities considered were absorbance averaged over the 226–2000 Hz band (AA2000, as measured in the Titan system and displayed as the peak value of the so-called wideband tympanogram), FR, and SC.

Based on the experimentally determined statistical distributions of test results for the Normal and Otosclerotic groups, we calculated SEN and SPE characteristics of tests based on each criterion. As seen in Figure 9, the test based on AA650 gave SEN and SPE values above 0.85 (for a normative AA650 of approximately 0.38). Estimated sensitivity and specificity of tests based on other measures were lower: about 0.8 for AA2000 and 0.55 for FR.

Application of SC to tests for otosclerosis needs to be specifically addressed. Theoretically, based on the distributions of the examined groups, one can expect a sensitivity and specificity of approximately 0.75 when SC is chosen as the test criterion (for a normative SC ≈ 0.46 mL). However, it needs to be remembered that, following what is generally assumed in audiology, the criterion for inclusion to the norm group required SC should be ≥0.3 mL [9,30]. Therefore, all cases of SC > 0.3 should be classified as normal rather than pathological, meaning that in our Otosclerotic group a test for otosclerosis based on the SC criterion would have a sensitivity of about 0.46 (and, of course, a 100% specificity). Such a test is practically useless, a statement consistent with the conclusions of other researchers [11,14,30].

FR seems to be the measure least suitable for an otosclerosis test. In theory, otosclerosis should cause a significant increase in FR due to an increase in stiffness of the ossicular chain. However, the observed variability of FR is large, again consistent with observations of others [44]. Although the average FR is higher in otosclerotic ears than in normal ears, the distributions of FR values overlap considerably. It is difficult to discern the reasons for such variability. Along with intersubject differences, it could be due to measurement errors in the system. There is no information available on how FR is calculated in the Titan software. Thus, we cannot exclude the possibility that the internal procedure used for evaluating FR is inappropriate. Notwithstanding, the sensitivity and specificity of any otosclerosis test based on FR is low (about 0.55) and the corresponding AUC is about 0.67, which is only on the verge of significance.

Although the sensitivity and specificity of an otosclerosis test based on some absorbance-related measure may be relatively high (above 85%), one may still need to improve it. Theoretically, it is possible to apply a test battery consisting of multiple tests based on different absorbance-related measures. However, this is probably not practical, because all the above-described measures (AA650, AA2000, SC, and FR) are strongly correlated (Table 5). This means that sensitivity and specificity cannot be significantly improved by making a battery of such tests [33].

## 5. Concluding Remarks

It is generally agreed that testing energy absorbance of the middle ear can provide useful information about its status and facilitate detection of ear pathology. However, the applicability and reliability of these methods is still controversial. Our investigation focused on examining possible ways of using immittance-related tests to diagnose otosclerosis. Considering the number of otosclerotic patients who qualify for stapedotomy (and other reconstructive surgeries) in our clinic, any reliable objective diagnostic method which permitted early detection of otosclerotic changes would be particularly useful.

The investigation here has shown that a reasonably accurate method of testing for the presence of otosclerosis can be created based on energy absorbance measurements obtained with a standard, commercially available instrument.

The most suitable quantity for this purpose is the energy absorbance measured at tympanometric peak pressure for low frequencies, a region where otosclerotic changes readily affect mechanoacoustic properties of the middle ear. We found that the optimal measure to use as a test criterion is the average absorbance over the band of about 550 to 750 Hz. Our results demonstrate that, using this measure, one can achieve high sensitivity and specificity (above 85%) and high accuracy, described with an AUC of greater than 0.9. The “wideband tympanogram” measure, which is implemented in the current commercial WBT system, can also be used, but it is less effective. There seems to be very limited usefulness of other immittance-related measures such as SC and FR.

An unexpected finding in this study was the effect of gender in the group of otosclerotic patients, which appeared in the higher frequency range. This suggests that gender might be considered as a variable in future studies on immittance measures in otosclerosis.

Our experience, and the opinions of other researchers, is that reasonably accurate absorbance measurements can be achieved with commercial systems. However, it needs careful verification of system functioning, frequent and precise calibration (more precise than provided in standard maintenance), and measures to limit random errors (e.g., adequate control over probe insertion, ensuring good cooperation with the patient, and so on).

Further investigations are needed to refine the method. Among important problems, one could evaluate postoperative changes in middle ear absorbance and relate them to audiological effects. Another interesting field of investigation would be patients undergoing other types of ear surgery, including cochlear and middle ear implants.

## Figures and Tables

**Table 1:** Age, gender, and ear side of investigated groups.

Group	Normal			Otosclerotic		
Gender	Male	Female	Total	Male	Female	Total
No of ears	38	88	126	25	65	90
No of ears: right/left	19/19	43/45	62/64	14/11	34/31	48/42
Mean age (year)	33.7	28.8	30.3	41.5	44.5	44.2
SD age (year)	7.7	6.3	7.2	11.7	9.4	9.9
Maximum age (year)	51.1	56.9	56.9	66.2	68.0	68.0
Minimum age (year)	18.0	18.5	18.0	11.9	14.6	11.9

**Table 2:** Results of ANOVA. Effects of the independent variables Group, Ear, and Gender and dependent repeated-measure variable frequency (FREQ) on absorbance.

Effect	SS	df	MS	*F*	*p*	Eta-Squared
Free component	804.0796	1	804.0796	9209.009	<0.001	0.977912
Group	10.4048	1	10.4048	119.164	<0.001	0.364234
Gender	0.1799	1	0.1799	2.060	0.152723	0.009806
Ear	0.0312	1	0.0312	0.357	0.550601	0.001715
Group*Gender	0.5563	1	0.5563	6.371	0.012348	0.029719
Group*Ear	0.1344	1	0.1344	1.539	0.216118	0.007346
Gender*Ear	0.1127	1	0.1127	1.290	0.257306	0.006165
Group*Gender*Ear	0.1082	1	0.1082	1.240	0.266846	0.005924
Error	18.1614	208	0.0873			
FREQ	118.0758	21	5.6227	378.967	<0.001	0.645636
FREQ*Group	3.6076	21	0.1718	11.579	<0.001	0.052731
FREQ*Gender	0.8214	21	0.0391	2.636	<0.001	0.012517
FREQ*Ear	0.1894	21	0.0090	0.608	0.916375	0.002914
FREQ*Group*Gender	0.4527	21	0.0216	1.453	0.082955	0.006936
FREQ*Group*Ear	0.1604	21	0.0076	0.515	0.966171	0.002469
FREQ*Gender*Ear	0.0348	21	0.0017	0.112	1.000000	0.000537
FREQ*Group*Gender*Ear	0.1932	21	0.0092	0.620	0.907627	0.002972
Error	64.8072	4368	0.0148			

Key: SS—Sum of squares (total), df—Degree of freedom, MS—Mean square, *F*—*F*-test statistic, *p*—Significance level, and Eta-squared (η^2^)—Effect size.

**Table 3:** Results of Mann–Whitney U-test for comparing absorbance between the Normal and Otosclerotic groups.

Variable	Sum Rank Norm	Sum Rank Oto	*U*	*Z*	*p*	N Valid Norm	N Valid Oto	AUC
AA241	17,406	6030	1935	8.247	<0.001	126	90	0.829
AA288	16,687	6750	2655	6.658	<0.001	126	90	0.766
AA343	17,176	6261	2166	7.738	<0.001	126	90	0.809
AA408	17,855	5581	1486	9.238	<0.001	126	90	0.869
AA485	18,001	5435	1340	9.561	<0.001	126	90	0.882
AA581	18,275	5161	1066	10.166	<0.001	126	90	0.906
AA696	18,208	5228	1133	10.018	<0.001	126	90	0.900
AA828	17,781	5655	1560	9.075	<0.001	126	90	0.862
AA985	17,011	6425	2330	7.375	<0.001	126	90	0.795
AA1172	16,462	6974	2879	6.162	<0.001	126	90	0.746
AA1393	15,269	8167	4072	3.528	<0.001	126	90	0.641
AA1657	15,352	8085	3990	3.710	<0.001	126	90	0.648
AA1971	14,932	8504	4409	2.784	0.005	126	90	0.611
AA2344	14,836	8600	4505	2.572	0.010	126	90	0.603
AA2787	14,707	8729	4634	2.287	0.022	126	90	0.591
AA3315	14,904	8532	4437	2.722	0.006	126	90	0.609
AA3942	14,642	8794	4699	2.143	0.032	126	90	0.586
AA4688	13,679	9758	5663	0.015	0.988	126	90	0.501
AA5575	13,758	9679	5584	0.190	0.849	126	90	0.508
AA6630	13,859	9577	5482	0.414	0.679	126	90	0.517
AA7660	14,785	8652	4557	2.458	0.014	126	90	0.598
AA2000	17,759	5677	1582	9.026	<0.001	126	90	0.860

Key: Sum.rank Norm and Sum.rank Oto—Sum of ranks in the Normal and Otosclerotic groups, respectively, *U*—The smaller of two U-Statistics, *Z*—Standardized value of normally-distributed variable, *p*—Significance level, and AUC—Area under ROC curve.

**Table 4:** Results of Mann–Whitney U-test for selected immittance-related measures.

Variable	Sum Rank Norm	Sum Rank Oto	U	Z	*p*	N Valid Norm	N Valid Oto	AUC
AA650	18,273	5163	1068	10.161	<0.001	126	90	0.906
AA2000	17,759	5677	1582	9.026	<0.001	126	90	0.860
SC	19,381	6270	2265	7.977	<0.001	137	89	0.814
FR	13,004	12,874	3551	−5.400	<0.001	137	90	0.712

AA650: average absorbance over the 545–749 Hz band (the frequency interval encompassing the range of maximum AUC); AA2000: average absorbance over the 226–2000 Hz band, routinely measured in the Titan WBT system (when “adult” program option is chosen); SC: “static compliance”, actually the absolute value of middle ear admittance measured at tympanometric peak pressure (TPP) with a 226 Hz tone; and FR: middle ear resonance frequency.

**Table 5:** Cross-correlation coefficients (*r*) and related *p*-values between absorbance-related measures in the Normal and Otosclerotic groups.

Normal					Otosclerotic				
Variable	AA650	AA2000	FR	SC	Variable	AA650	AA2000	FR	SC
AA650	r = 1.000	r = 0.7841	r = −0.5278	r = 0.7716	AA650	r = 1.000	r = 0.9006	r = −0.3543	r = 0.6772
	*p* = ---	*p* < 0.001	*p* < 0.001	*p* < 0.001		*p* = ---	*p* < 0.001	*p* = 0.001	*p* < 0.001
AA2000	r = 0.7841	r = 1.000	r = −0.2256	r = 0.6627	AA2000	r = 0.9006	r = 1.000	r = −0.2663	r = 0.6217
	*p* < 0.001	*p* = ---	*p* = 0.012	*p* < 0.001		*p* < 0.001	*p* = ---	*p* = 0.013	*p* < 0.001
FR	r = −0.5278	r = −0.2256	r = 1.0000	r = −0.4774	FR	r = −0.3543	r = −0.2663	r = 1.000	r = −0.2783
	*p* < 0.001	*p* = 0.012	*p* = ---	*p* < 0.001		*p* < 0.001	*p* = 0.013	*p* = ---	*p* = 0.009
SC	r = 0.7716	r = 0.6627	r = −0.4774	r = 1.000	SC	r = 0.6772	r = 0.6217	r = −0.2783	r = 1.000
	*p* < 0.001	*p* < 0.001	*p* < 0.001	*p* = ---		*p* < 0.001	*p* < 0.001	*p* = 0.009	*p* = ---

AA650: average absorbance over the 545–749 Hz band (the frequency interval encompassing the range of maximum AUC); AA2000: average absorbance over the 226–2000 Hz band, routinely measured in the Titan WBT system (when “adult” program option is chosen); SC: “static compliance”, actually the absolute value of middle ear admittance measured at tympanometric peak pressure (TPP) with a 226 Hz tone; and FR: middle ear resonance frequency.
